# Histone Methylation Analysis and Pathway Predictions in Chickens after MDV Infection

**DOI:** 10.1371/journal.pone.0041849

**Published:** 2012-07-26

**Authors:** Juan Luo, Apratim Mitra, Fei Tian, Shuang Chang, Huanmin Zhang, Kairong Cui, Ying Yu, Keji Zhao, Jiuzhou Song

**Affiliations:** 1 Department of Animal and Avian Sciences, University of Maryland, College Park, Maryland, United States of America; 2 United States Department of Agriculture, ARS, Avian Disease and Oncology Laboratory, East Lansing, Michigan, United States of America; 3 Laboratory of Molecular Immunology, National Heart, Lung and Blood Institute, National Institutes of Health, Bethesda, Maryland, United States of America; 4 Department of Animal Science, Michigan State University, East Lansing, Michigan, United States of America; University of California, Davis, United States of America

## Abstract

Marek's disease (MD) is a lymphoproliferative disease in chicken induced by Marek's disease virus (MDV). Although studies have focused on the genetic differences between the resistant and susceptible chicken, less is known about the role of epigenetic factors in MD. In this study, genome-wide histone modifications in the non-MHC-associated resistant and susceptible chicken lines were examined. We found that tri-methylation at histone H3 Lys4 (H3K4me3) enrichment is positively correlated with the expression of protein coding genes as well as microRNA (miRNA) genes, whereas tri-methylation at histone H3 Lys27 (H3K27me3) exhibits a negative correlation. By identifying line-specific histone modifications in MDV infection, we found unique H3K4me3 islands in the resistant chicken activated genes, which are related to immune response and cell adhesion. Interestingly, we also found some miRNAs from unique H3K27me3 patterns in the susceptible chickens that targeted genes involved in 5-hydroxytryptamine (5-HT)-receptor and adrenergic receptor pathways. In conclusion, dynamic line-specific histone modifications in response to MDV infection suggested that intrinsic epigenetic mechanisms may play a role in MD-resistance and -susceptibility.

## Introduction

Marek's disease (MD) is a lymphoproliferative disease characterized by infiltration of proliferating lymphoid cells into many tissues, including peripheral nerves, skin, muscle, liver, spleen, heart, kidney, gonads and proventriculus [1]. The causative agent of MD is Marek's disease virus (MDV) which is a cell-associated herpesvirus belonging to a subgroup of Alphaherpesviridae according to its genome organization [2], [3]. This disease is also a unique natural model for lymphomas that overexpress Hodgkin's antigen (CD30) in humans [4]. The pathogenesis of MDV in susceptible chickens can be divided into four phases [5]. First, there is an early cytolytic phase (2–7 days post-infection, dpi) primarily in B lymphocytes which results in lymphocytolysis and inflammation. Subsequently, the viruses activate CD4^+^ T cells to start a latent infection phase characterized by the lack of expressed viral antigens and infectious virus particles from 7 dpi onwards. A late cytolytic phase with the reactivation of virus is observed after 18 dpi. Finally, infected lymphocytes are transformed and proliferate profusely from 28 dpi onwards to form tumors in various tissues.

Chickens differ in their response to infection, due, in part, to genetic resistance to MD (lymphoma development) [6], [7], [8], [9]. The breeding of genetically resistant chicken is being considered as a new strategy to control MD. Importantly, both major histocompatibility complex (MHC)-associated and non-MHC associated resistance to MD have been bred [1]. In this study, we used the non-MHC associated resistant (L6_3_) and susceptible (L7_2_) chickens developed and maintained in USDA-ARS Avian Disease and Oncology Laboratory (ADOL) at East Lansing, Michigan [10]. Since the characterization of these two chicken lines, many studies have been conducted to understand the mechanism of MD-resistance in these chickens. Many candidate genes were found to be differential expressed in response to MDV infection [11], [12], [13]. Association studies revealed that polymorphisms in the *growth hormone* (*GH1*) gene were associated with MD resistance [14], [15]. Using genomic mapping methods, several quantitative trait loci linked to MD resistance were mapped on chromosomes 1–5, 7–9, 15, 18, 26, Z, E21 and E16 [16], [17]. Despite these initial studies from the genetic viewpoint, the mechanism underlying MD resistance in these chickens is still unclear.

Tumorigenesis is induced by complex procedures including both genetic and epigenetic factors [18], [19]. And epigenetic modifications on DNA and histones are considered to play an important role in tumor progression [20]. Notably, in our prior study we found that MD-resistant and susceptible birds have different DNA methylation levels on several candidate genes, indicating the potential functions of epigenetic factors in inducing different tumor incidence rates [21], [22], [23]. However, nothing is known about the histone modification patterns in these two chicken lines. In recent years, at least eight different modifications have been found on the unstructured N-terminal “tail” of core histones [24], with most of them being histone acetylation and methylation. By using chromatin immunoprecipitation combined with microarray (ChIP-Chip) or massively parallel sequencing (ChIP-Seq), genome-wide maps of histone marks have been generated in plant [25], [26], yeast [27], *C. elegans* [28], human [26], [29], [30], mice [31], [32] and zebrafish [33]. It was found that histone acetylations, such as acetylation at H3 Lys9 (H3K9ac) and Lys14 (H3K14ac), are related to active gene expression in human cells [34], [35]. Meanwhile, some histone methylations such as mono-, di-, and tri-methylation at histone H3 Lys4 (H3K4me1, H3K4me2, H3K4me3) are associated with gene activation, whereas others such as tri-methylation at H3 Lys9 (H3K9me3) and Lys27 (H3K27me3) are related to gene silencing [36], [37], [38]. It is worth noting that global differences in lysine methylation levels of histone have been identified between normal and cancerous tissues, indicating the alteration of histone modification pathways in cancer [39], [40], [41], [42]. Unfortunately, little is known about the detailed mechanism of histone modifications in tumorigenesis.

To gain more insight into the function of histone modifications in MD, we performed a histone landscape analysis using ChIP-Seq in the unique MD-resistant (L6_3_) and –susceptible (L7_2_) chicken lines both before and after MDV infection. Since the MDV induced changes are mainly initiated at the latent phase of infection [43], [44], we generated genome-wide maps of H3K4me3 and H3K27me3 in the spleen of these chicken lines at this stage. From this study, we found large number of line-specific H3K4me3 modifications, and found that their underlying genes are enriched in immune response and cell adhesion in L6_3_ chicken. Interestingly, we also found that the virus-induced specific H3K27me3 patterns in L7_2_ chicken overlapped with some miRNAs which target genes involved in novel pathways that may be related to MD-susceptibility.

## Results

### Line-specific H3K4me3 and H3K27me3 maps in chicken with varying resistance to MD

To determine the histone methylation profiles and its relationship with MD, we generated genome-wide H3K4me3 and H3K27me3 maps in L6_3_ and L7_2_ chickens in an MDV challenge experiment (See Figure S1 in the supplemental material). By using SICER [32], a commonly used analysis algorithm, we found 14,798 H3K4me3 islands and 22,503 H3K27me3 non-overlapping islands across the four groups of chickens. A comparable number of H3K4me3 islands were found among these four groups (Table 1). In the non-infected samples, more H3K4me3 islands were found in L7_2_ compared to L6_3_; while after MDV infection, the number of H3K4me3 islands increased slightly in L6_3_. However, the number of H3K27me3 islands was highly variable (Table 1), with the smallest in L6_3_ infected samples, the largest in 7_2_ infected samples and the intermediate in 7_2_ non-infected samples and 6_3_ non-infected samples. Most of the H3K4me3 islands overlapped in different chickens, with less than 5% unique in each group. Interestingly, fewer overlaps were observed in case of H3K27me3.

**Table 1:** Distribution of H3K4me3 and H3K27me3 islands in each group.

	H3K4me3				H3K27me3			
Sample	Total islands	Genes	Unique islands	Unique genes	Total islands	Genes	Unique islands	Unique genes
63.Non	11829	8131	422	30	8403	2885	1097	118
63.Inf	13068	8089	636	54	5327	2513	589	144
72.Non	12948	9264	635	40	14035	4713	2332	266
72.Inf	11163	9107	231	20	17906	5117	5659	592

**Figure 1:**
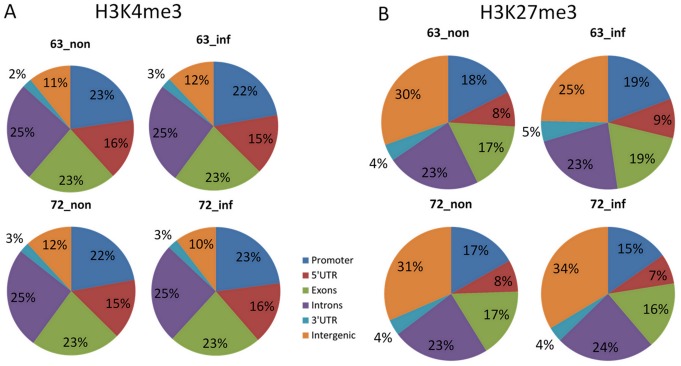
Percentage of the H3K4me3 and H3K27me3 islands distribution on genes. The chicken genome was divided into six parts: the promoter region, 5′ UTR, intron, exon, 3′ UTR and intergenic region. The histone methylation islands were mapped to these genomic regions and the percentage of these mapped islands were calculated. 63_non: Line 63 non-infected group; 63_inf: Line 63 infected group; 72_non: Line 72 non-infected group; Line 72 infected group. Percentage of the H3K4me3 (A) and H3K27me3 (B) distribution on genes.

**Figure 2:**
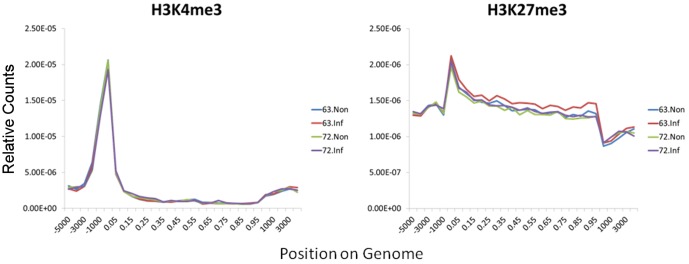
H3K4me3 and H3K27me3 enrichment density along chicken genome. The enrichments of H3K4me3 and H3K27me3 modifications were respectively plotted around TSS (transcription start site), promoter region (from -5000 to TSS), the gene body region, TTS (transcription termination site) and intergenic region (from 3000 bp to TTS). We scaled a percentage of length instead of the true length to do the plotting to solve the problem of genes with different exon and intron length.

To obtain an overall picture of H3K4me3 and H3K27me3 distributions along the chicken genome, we divided the chicken genome into six regions: promoter, 5′ UTR, exon, intron and 3′ UTR of genes and intergenic regions. As shown in Figure 1A, although most of the H3K4me3 islands were found in promoter, exon, and intron, the density was much higher in the promoter region (Figure 2). Similar distributions of H3K4me3 islands were found among the chickens, with no more than 1% differences (Figure 1A). Most of the H3K27me3 islands were found in intergenic, exon, intron and also the promoter region (Figure 1B), with a higher density in the promoter and gene body (Figure 2). Different chickens have different distributions of H3K27me3 islands with the largest differences found in the intergenic region especially after MDV infection (Figure 1B). The H3K27me3 occupancy in intergenic region decreased in L6_3_ from 30% to 25% after infection, however it increased in L7_2_ from 31% to 34%.

**Figure 3:**
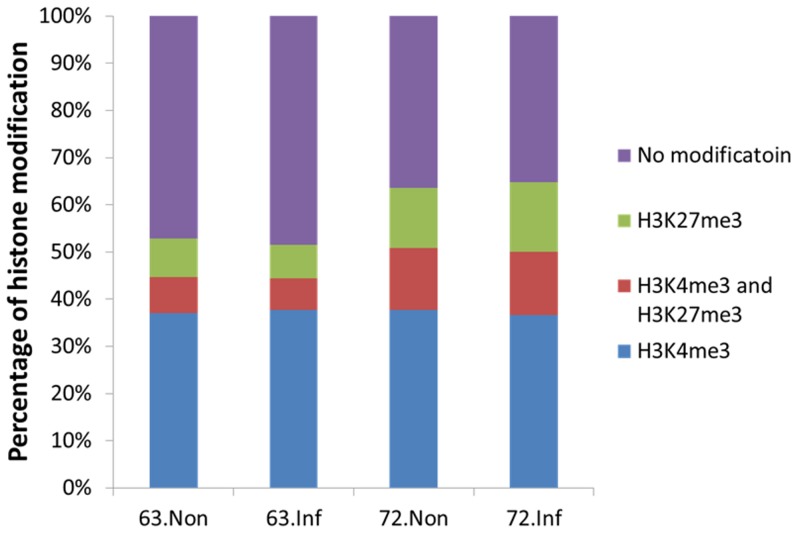
Percentage of genes with only H3K4me3, or only H3K27me3 or both H3K4me3 and H3K27me3 enrichment in the chicken genome for each group. When there is an overlap of the H3K4me3 island with the TSS of a gene, it was defined as the H3K4me3 enrichment on this gene. For H3K27me3, the enrichment was defined as the overlap with the gene body of a gene.

In the chicken genome, more than 50% of the genes are marked by H3K4me3 and H3K27me3 (Figure 3). The genes marked by histone methylation can be divided into three categories: genes only having the H3K4me3 mark (K4 only), genes only having H3K27me3 mark (K27 only), and genes with both H3K4me3 and H3K27me3 marks (bivalent). Interestingly, most of the genes in all chickens are K4 only (ranging from 37% to 38%). In non-infected samples, more K27 only and bivalent genes were found in L7_2_ (Figure 3). No big differences of the number of K4 only, K27 only and bivalent genes were found before and after MDV infection within lines.

### Correlation between histone methylation levels and gene expression across different chicken lines and different treatments

As H3K4me3 and H3K27me3 were considered active and repressive marks respectively [36], [37], [38], we subsequently checked how the histone methylation profiles were associated with the gene expression, especially the change of gene expression induced by MDV infection.

**Figure 4:**
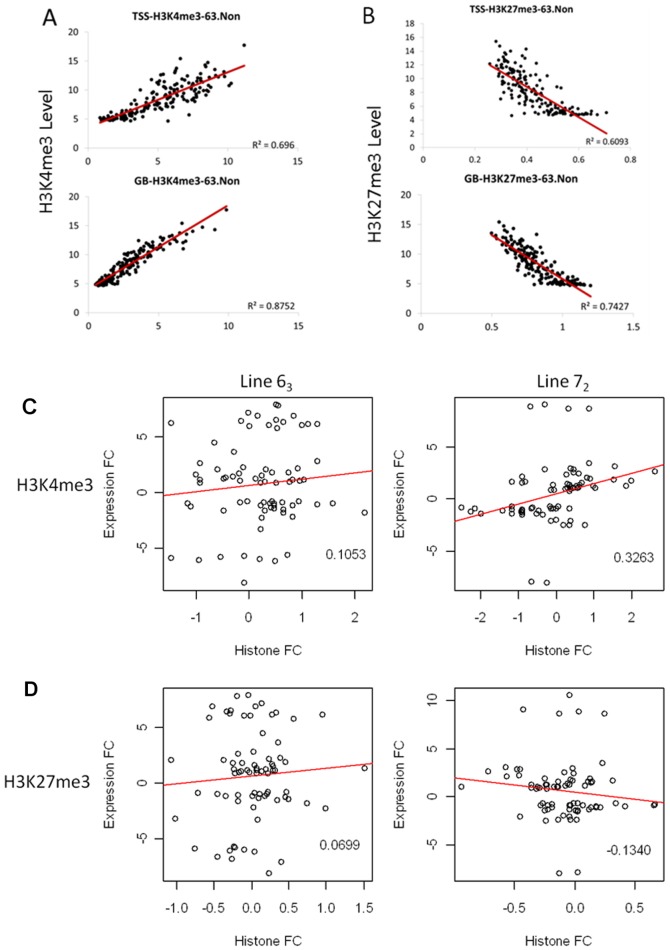
Correlation of histone modification enrichment and gene expression in TSS and gene body (GB) region. A. Correlation of H3K4me3 enrichment and gene expression at both TSS and GB region. Genes were grouped into 50 gene-set (1 dot in the figure) according to their expression level. The same set of genes was used to do the correlation analysis both in TSS and GB region.The Y-axis is the histone methylation level and X-axis is the expression level with a Log_2_ transformation. The result of L63 noninfected sample were shown as an example. B. Correlation of H3K27me3 enrichment and gene expression at both TSS and GB region. The result of L63 noninfected sample were shown as an example. C. The correlation between MDV induced H3K4me3 enrichment change and gene expression change. The numbers within the individual plots correspond to the Pearson correlation coefficient (rho). Histone FC: MDV induced histone modification fold change; Expression FC: MDV induced mRNA expression fold change. D. The correlation between MDV induced H3K27me3 enrichment change and gene expression change.

By plotting the H3K4me3 and H3K27me3 enrichments along the genome, we found that for the genes with H3K4me3 enrichment, when the enrichment level is high, the expression of these genes is high as well (See Figure S2 in the supplemental material). However, it is opposite for genes with H3K27me3 enrichment (See Figure S3 in the supplemental material). By quantifying the amount of H3K4me3 and H3K27me3 in the transcription start site (TSS) and gene body (GB) regions and the expression of related genes, we found that regardless of MDV infection the H3K4me3 level showed a positive correlation (R^2^ ranging from 0.6581 to 0.8752) and H3K27me3 showed a negative correlation (R^2^ ranging from 0.4672 to 0.7427) with gene expression level in both chicken lines (Figure 4A, B and Figure S4).

However, relationship between the histone modification change and gene expression change is a little complicated. We first checked several candidate genes, including *CD8α*, *CTLA4* and *IL8* from the MD-resistant and –susceptible gene list we identified in previous research [43]. We found that when the histone modification changed, the gene expression changed accordingly in both chicken lines (Figure S5). Taking an example of CD8α, we can see that the number of reads in a H3K4me3 island located on the second exon is much greater in the L6_3_ infected sample than in the non-infected sample. Correspondingly, the expression of this gene is significantly up-regulated after MDV infection. On the other hand, the reads only have a slight increase in the L7_2_ infected sample compared with the non-infected sample which is consistent with the slight increase of the expression level. Global correlation analysis revealed a slight positive correlation between the H3K4me3 change and the mRNA expression change induced by MDV both in L6_3_ and L7_2_ with a higher correlation shown in L7_2_ (Figure 4C). For H3K27me3, almost no correlation was detected in L6_3_, whereas a slight negative correlation was found in L7_2_ (Figure 4D).

In summary, irrespective of the chicken line and MDV infection, gene expression levels were correlated with H3K4me3 and H3K27me3 marks in a positive or negative manner, respectively. However, the MDV induced histone modification varied in their ability to regulate gene expression. A better correlation between the histone modification change and the expression change was detected in L7_2_ compared to L6_3_.

### Functional analysis of genes with unique histone modifications

**Figure 5:**
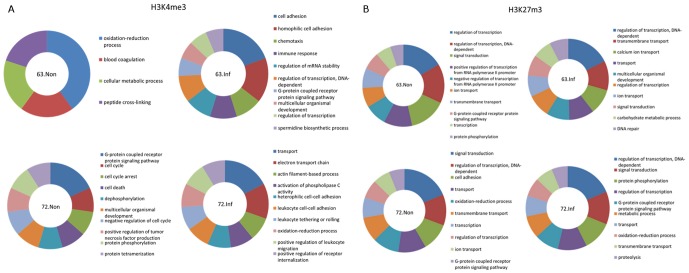
Gene functional analysis of the genes with unique histone modification islands in different groups. A. Gene functional analysis of the genes with unique H3K4me3 islands by IPA. Except for Line 6_3_ non-infected group, 10 enriched pathways were shown in figure. Larger size of the sector means this pathway is represented by more genes. 63.Non: Line 6_3_ non-infected group; 63.Inf: Line 6_3_ infected group; 72.Non: Line 7_2_ non-infected group; 72.Inf: Line 7_2_ infected group. B. Gene functional analysis of the genes with unique H3K27me3 islands.

To further explore the implication of the histone marks in MD resistance, we separated the histone methylation islands into two categories: (a) universal islands which can be found in all four groups and (b) unique islands which were detected in one group. In our experiment, we found 422, 636, 635 and 231 unique H3K4me3 islands and 1097, 589, 2332 and 5659 unique H3K27me3 islands in the L6_3_ non-infected, L6_3_ infected, L7_2_ non-infected and L7_2_ infected samples respectively (Table 1). After annotation of the histone marks with underlying genes, the genes mapping to the unique histone marks (See Table S1 in the supplemental material) were used to do pathway analysis using gene ontology (GO) terms to find the possible enrichment of bio-functions (Table S2). As shown in Figure 5A, the genes uniquely marked by H3K4me3 were enriched for different bio-functions between L6_3_ and L7_2_ in response to MDV infection. In L6_3_ non-infected group, genes were enriched in oxidation-reduction, blood coagulation and cellular metabolism (Figure 5A). In L7_2_ non-infected group, genes were enriched in cellular processes such as G-protein coupled receptor signaling pathways, cell cycle regulation and cell cycle arrest. On the other hand, genes involved in cell adhesion, homophilic cell adhesion, chemotaxis and immune response were activated in L6_3_ infected chickens while genes involved in transport, the electron transport chain, actin filament-based processes and activation of phospholipase C activity were activated in L7_2_ infected chickens (Figure 5A).

However, a similar set of genes were uniquely marked by H3K27me3 in L6_3_ and L7_2_ after MDV infection (Figure 5B). Genes enriched in regulation of transcription, signal transduction, transmembrane transport, ion transport and G-protein coupled receptor signaling pathways were found in both chicken lines before MDV infection while after MDV infection genes involved in regulation of transcription, transmembrane transport, signal transduction, and transport were detected in both L6_3_ and L7_2_.

### Histone modifications target microRNAs and regulate their expression

**Figure 6:**
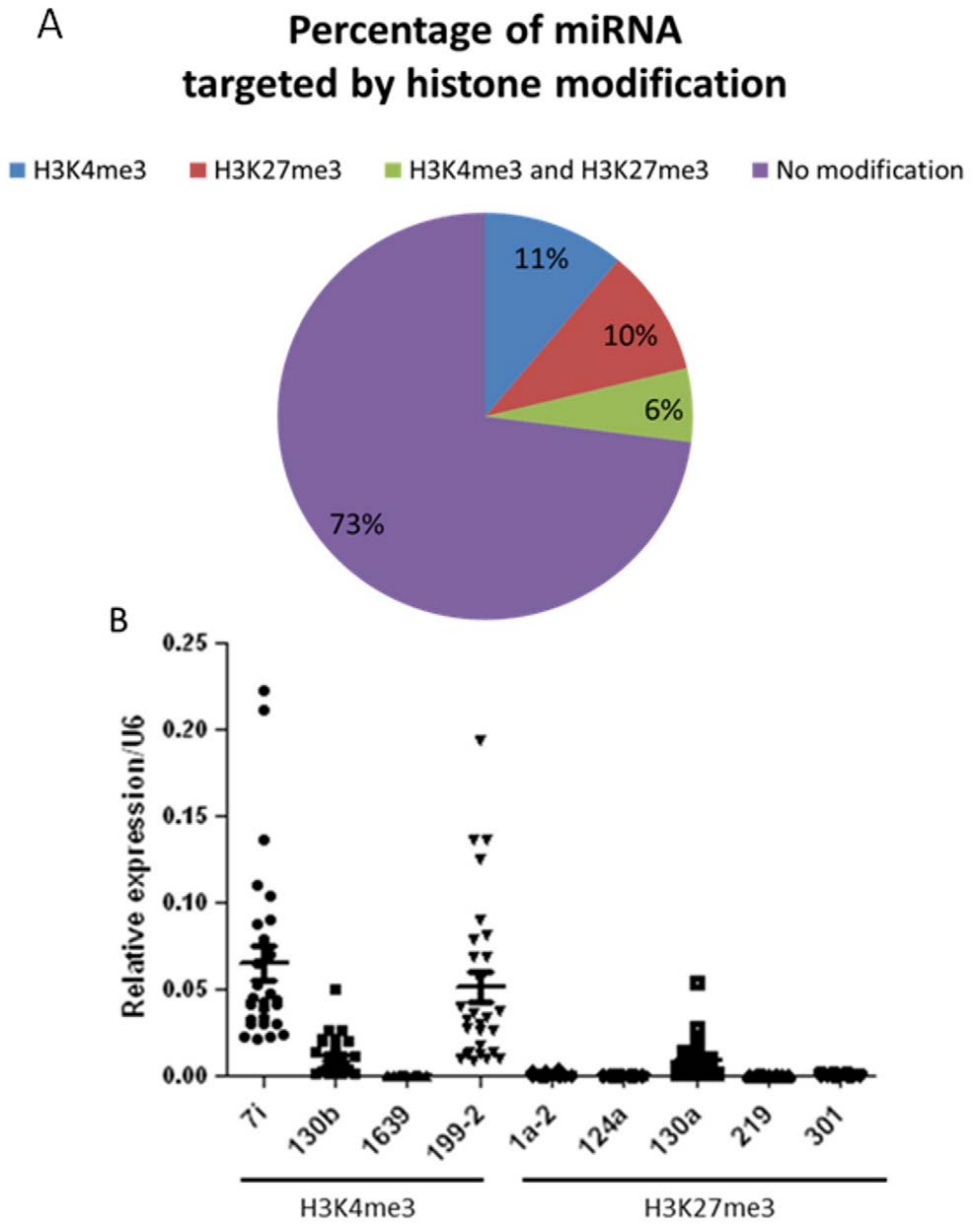
Histone modification targeted miRNAs and regulate their expression. A. The percentage of miRNAs that are targeted by histone modifications. B. The expression level of miRNAs marked by H3K4me3 and H3K27me3. The expression of miRNAs were detected by real-time quantitative PCR and normalized to U6. 7i: gga-let-7i, 130b:gga-mir-130b, 1639: gga-mir-1639, 199-2: gga-mir-199-2, 1a-2: gga-mir-1a-2, 124a: gga-mir-124a, 130a: gga-mir-130a, 219: gga-mir-219, 301: gga-mir-301.

Besides the protein coding genes, we also found 134 microRNAs (miRNA) that were targeted by H3K4me3 and H3K27me3 (See Table S3 and S4 in the supplemental material). Similar to protein coding genes, some miRNAs have only H3K4me3 (10%) or H3K27me3 (11%) marks while some are marked by both (6%) (Figure 6A). The number of miRNAs with either modifications is similar between H3K4me3 (55) and H3K27me3 (50). Different from protein coding genes, only 27% of the miRNAs are marked by histone modifications (Figure 6A).

As the expression of miRNAs has been reported to be regulated by histone modifications [45], [46], [47], we tested the relationships between miRNA expression and histone modifications. As demonstrated in Figure 6B, most of the miRNAs with H3K4me3 modifications have a much higher expression than those with H3K27me3 modifications. Also, the miRNA expression is positively correlated with H3K4me3 levels (R^2^ = 0.466 and Figure S6A). However the relationship between miRNAs expression and H3K27me3 level is more complex. When we considered the miRNAs marked by H3K27me3 as whole, we did not find any correlation between the expression and H3K27me3 levels; but for some individual miRNA, such as gga-mir-124a and gga-mir-310, we found very good negative correlation between the expression and H3K27me3 level (R^2^ = 0.7773 and 0.6632 respectively, and Figure S6B).

### Pathway analysis of unique miRNA targets marked by H3K27me3

**Figure 7:**
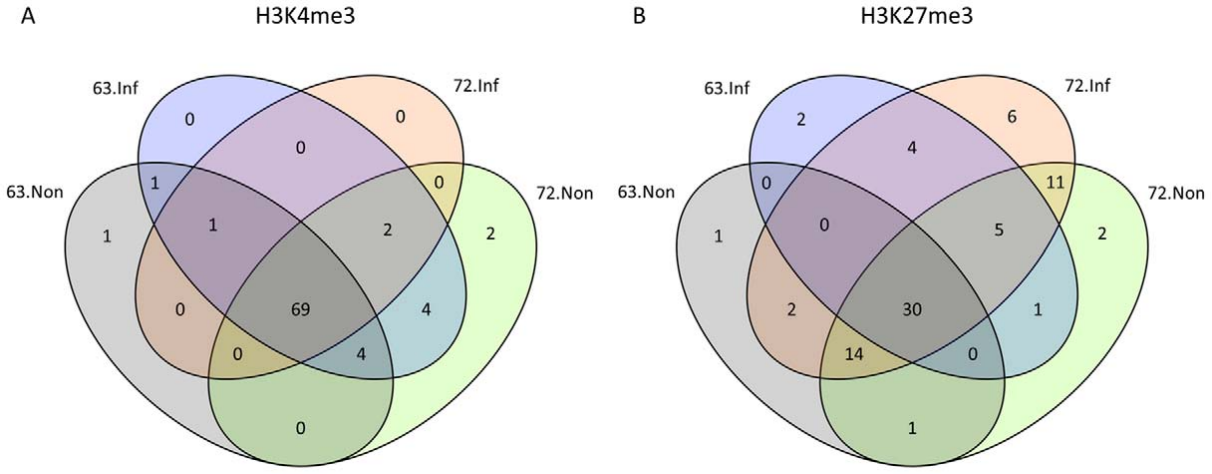
Venn Diagram of the number of miRNAs that are targeted by histone modifications. A. H3K4me3. B. H3K27me3.

We examined the miRNAs targeted by histone modifications in each group and plotted a Venn diagram to show the shared and unique miRNAs in each group (Figure 7). In case of H3K4me3, 69 out of 84 miRNAs were shared by all four groups (Figure 7A). However only 30 out of 79 miRNAs marked by H3K27me3 were shared by the four groups (Figure 7B). A dynamic histone modification pattern was apparent in both MD-resistant and –susceptible chicken lines. Compared with the non-infected group, the number of miRNAs marked by H3K4me3 increased in L6_3_ but decreased in L7_2_ (Figure 7A). In contrast, the number of miRNAs marked by H3K27me3 was increased in L7_2_ but decreased in L6_3_ (Figure 7B).

**Figure 8:**
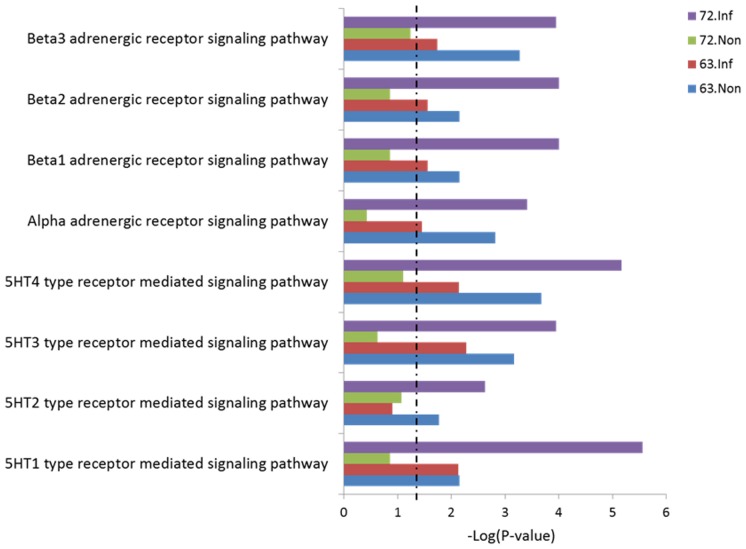
Pathway analysis of the targets genes of miRNA with unique H3K27me3 enrichment. The miRNA targets were classified in PANTHER (http://www.pantherdb.org/). Only pathways involved in 5-HT receptor pathway and adrenergic receptor pathway were shown. Dashed line: threshold line corresponds to P value of 0.05.

To further understand the potential biofunctions of the miRNAs, we used software tools to predict the targets of unique miRNAs marked by histone modifications and analyze the associated pathways. Interestingly, we found that a lot of pathways involving the 5-HT (5- hydroxytryptamine) receptor and adrenergic receptor which belong to the G protein coupled receptor (GPCR) family were highly enriched in the L7_2_ infected group (See Table S5 in the supplemental material and Figure 8). While the enrichment of these pathways remained the same or was decreased in L6_3_ after MDV infection, it was increased in L7_2_ (Figure 8). A similar phenomenon was also detected in other G protein coupled receptors (See Figure S7 in the supplemental material).

## Discussion

In this study, we reported the first genome-wide histone modification maps of H3K4me3 and H3K27me3 in chicken spleen during the latent phase of MDV infection by using MD-resistant and -susceptible chickens. Spleen provides a suitable microenvironment for the MDV transformed CD4^+^ T cells to produce progressive MD in susceptible L7_2_ chicken. The transformed cells are cleared after the cytolytic phase in resistant L6_3_ chicken, although the number of the transformed cells at the cytolytic phase is similar in the two lines [48], [49], [50]. The MDV loads in spleen differ between lines 6 and 7 at the latent stage (10 dpi), which made it a critical time point for studying the tumor microenvironment differences between these two chicken lines ([13], [44]).

### The function of histone modifications is conserved among species

We found more than 10,000 H3K4me3 islands in chicken; this value is less than that in the mouse genome [31], which contains more than 15,000 islands, and also the human genome, which contains approximately 17,000 islands [51]. This can be explained by different tissue or cell types we used from other studies. Since H3K4me3 marks the active genes or the bivalent genes that have the potential to be activated [32], the H3K4me3 islands are indicative of the number of activated or potentially activated transcripts in the spleen of chicken. However, the number of H3K27me3 islands identified in chicken was approximately 22,000, which is roughly a quarter of the number found in mice [31]. The smaller number of H3K27me3 islands in the intergenic or the intronic regions of the chicken can be partly explained by the smaller size of the chicken genome (1.2 billion bp) compared to that of the mouse (2.6 billion bp) and the human (3.0 billion bp) genomes. We also found that the percentage of genes marked by histone modifications in chicken differed from the number in mice, particularly genes with bivalent and H3K27me3 marks (Figure 2A) [31]. The different number of genes that can be potentially activated in each species may explain why a similar number of genes were found in human, mouse and chicken, but the number of genes activated was different in each case. Additionally, there were large differences in the island distributions. While most of the H3K4me3 islands were found in promoter and intergenic regions in mice [31] and humans [51], in chickens, H3K4me3 were distributed not only in the promoter but also in exons and introns (Figure 2B). Approximately 23% of the H3K4me3 islands were found in exons in chicken, compared to only 2% in mice. In addition, more than 50% of the H3K27me3 islands were found in intergenic regions in mice [31] and humans [51], but only 30% in chickens. Furthermore, a large number of H3K27me3 islands were found in promoters (about 17%) and introns (about 23%) in chicken, which also differs from the results found in mice [31] and humans [51]. The genome-wide distributions of H3K4me3 and H3K27me3 around the TSS region have patterns similar to that in humans [36], mice [31] and plants [52], particularly around ±300 bp of the TSS, with the exception that the H3K27me3 distribution in intergenic regions is different in these species. Interestingly, there is an elevation of H3K27me3 enrichments at the TTS and intergenic regions in mice [31], but the distribution is more complex in chickens.

Comparing the H3K4me3 and H3K27me3 islands in our samples, we found that the distribution of H3K27me3 islands varied a lot in different groups. In human, histone-lysine N-methyltransferase 1 and 2 (EZH1 and EZH2) are responsible for H3K27me3 methylation and lysine (K)-specific demethylase 6A and 6B (KDM6A and KDM6B) are responsible for the demethylation of H3K27me3 [53]. The change of H3K27me3 enrichment may be due to the changes in activity of the methyltransferase or demethylase only, or both. Recently, a finding showed an increase of KDM6B in Hodgkin's Lymphoma caused by Epstein-Barr virus (EBV) which indicated that the demethylase in chicken may also be the main contributor to the change of H3K27me3 induced by MDV infection [54]. However, compared with human, less is known about the H3K27me3 methyltransferases and demethylases in chicken yet.

Coming back to the function of the histone methylations, it was found that H3K4me3 is positively correlated with gene expression, whereas H3K27me3 is negatively correlated with gene expression [36], [37], [38]. By combining the gene expression and histone modification profiles, we found the same phenomena. The H3K4me3 enrichment at the TSS region and GB is positively correlated with gene activation while the H3K27m3 has an inverse association with gene activation. This results are consistent with results in other species such as yeast [27], plants [52], [55], [56], mice [31], [32], and humans [36], [51] as well as in stem cell differentiation in humans [51]. In contrast, differences were found in correlation between the MDV induced histone modifications and gene expression changes. A better correlation was shown in L7_2_ in both H3K4me3 and H3K27me3 compared to L6_3_. Since L7_2_ chickens are more susceptible to MD, the result indicated that MDV changes the host expression through the change of histone modification in the susceptible chickens. Although histone modification levels changed in response to MDV infection even in the resistant chickens, the functional correlation between the two appears to be different.

All in all, despite the differences of the H3K4me3 and H3K27me3 islands distribution along the chicken genome from other species, the function of these two histone methylations is conserved across species with H3K4me3 enrichment positively correlated with gene expression and H3K27me3 enrichment negatively correlated with gene expression both at the TSS and GB region.

### Unique histone modification profiles induced by MDV predict novel pathways involved in MD-resistance and -susceptibility

Although the mechanism of MD resistance is not yet clear, many studies have demonstrated that immune genes are differently expressed between these two chicken lines in response to MDV infection [11], [12], [13], [15], [57] which is representative of the immunosupression in susceptible chickens. In our current research, we confirmed that genes involved in immune response may play role in MD-resistance by pathway analysis using genes with unique H3K4me3 islands. Although most of the genes are novel genes, we did find some genes that were shown in other publications, such as chemokine (C-C motif) ligand 1 (*CCL1*, ENSGALT00000003670) [58]. CCL1 is a chemokine secreted by monocytes, which can activate macrophages and T lymphocytes [59]. It was found that CCL1 is important for peritoneal adhesion which matched our finding of cell adhesion functions in L6_3_ infected sample [60]. We also found that the sterile alpha and TIR motif containing 1 (*SARM1*, ENSGALT00000005687) gene has a unique H3K4me3 island in L6_3_ infected sample. This gene encodes a Toll-IL-1 receptor adaptor belonging to the Myd88 family which is a negative regulator of the innate immune response [61], [62]. It was found that the SARM1 deficient mice were more susceptible to the West Nile virus (WNV) induced disease [63], which indicated the importance of SARM1 induction in resistance of virus induced disease. In addition, we also found that some genes related to cell adhesion were activated only in L6_3_, whereas some genes related to transport activated only in L7_2_ after MDV infection. In normal tissue, cell-cell adhesion plays important role in maintaining the structure of the tissue. However, reduced cell-cell adhesion allows the invasion and metastasis of cancer cells [64]. The activated cell adhesion genes could help L6_3_ chicken block the spread of transformed cells.

MiRNAs are a class of non-coding RNAs that are about 22 nucleotides long [65]. By negatively regulating gene expression at the post-transcriptional level, miRNAs play a role in several biological functions including apoptosis, cell proliferation, and tumorigenesis [65], [66], [67]. It was found in recent years that the expression of miRNAs can be regulated by chromatin structure [45], [46], [47], [68], which was also confirmed in our experiment. H3K4me3 as a positive marker of protein coding genes is also positively correlated with miRNA expression. Most of the miRNAs with H3K27me3 modification have a very low expression, which indicated a repressive effect of H3K27me3 on miRNAs.

Interestingly, by analyzing the target of miRNAs marked with H3K27me3, we found some novel pathways that may be involved in MD-susceptibility. 5-HT receptors are members of the GPCR family found in the nervous system that play important roles in several neurological processes such as memory, mood, learning and sleep [69]. Adrenergic receptors also belong to the GPCR family, and are targets of catecholamines (CAs) [70]. Both 5-HT and adrenergic receptor-mediated signaling pathways were found to be involved in the interaction between sympathetic nerve system (SNS) and immune system [71], [72], suggesting that the non-MHC associated MD-resistance may originate from the brain instead of the immune organs themselves. By releasing neurotransmitters and neuropeptides like CAs, SNS could alter the response of immune system through the neurotransmitter receptors, including 5-HT receptor and adrenergic receptors, presented on immunocytes [71]. CAs are known to function as immunosupressive agents by downregulating the proliferation and differentiation of immunocompetent cells [73], which might explain why an upregulation of the receptors for CAs was observed in MD-susceptible chickens exhibiting immunosuppression during latent infection [74]. In addition, the expression of higher levels of several cytokines in MD-susceptible chickens may also result from a higher level of CAs [13]. In our previous research, we found that the *NOS2* gene, which regulates the generation of NO (nitric oxide) [75], was up-regulated in the non-MHC-associated MD-susceptible chicken lines (L7_2_) [57], which is contrary to the finding in MHC-associated MD-susceptible chicken line (line S_13_ and line P2a) [76], [77]. Although it is unclear if increased *NOS2* would induce immunosuppression in L7_2_ chicken, we could speculate that the upregulation of *NOS2* was induced by signals from SNS, as 5-HT is a very important mediator for *NOS* activation [78]. Moreover, both 5-HT and CAs can stimulate the release of growth hormone [79], [80], which may explain the finding of a MD-resistant candidate, *GH1* gene [81].

Overall, this research provides the first genome-wide H3K4me3 and H3K27me3 maps in MD-resistant and –susceptible chickens before and after MDV infection. Like other species, such as human and mice, the H3K4me3 and H3K27me3 functions in gene expression are conserved. H3K4me3 enrichment is positively correlated with gene expression around the TSS and the gene body region, whereas H3K27me3 enrichment is negatively associated with gene expression. Line-specific dynamic changes of histone modifications were found between MD-resistant and –susceptible chicken. Functional analysis of the genes and miRNAs with unique histone modifications, as well as identifications of novel pathways, could provide new insights into decoding the mechanism of disease resistance both in animals and human.

## Materials and Methods

### Animals and viruses

Chickens from two specific-pathogen-free inbred lines of White Leghorn (L6_3_, resistant to MD and L7_2_, highly susceptible to MD), were hatched, reared, maintained and challenged in the Avian Disease and Oncology Laboratory (ADOL, Michigan, USDA). The experiment design of our study is shown in Figure S1A. We chose six chickens from each chicken line, inoculated 3 of them with MDV and 3 of them non-infected as age-matched control. The infected chickens were challenged intra-abdominally with a partially attenuated very virulent strain plus (vv+) MDV, 648A passage 40, at 5 days after hatch with a viral dosage of 500 plaque-forming units (PFU) per bird. The chickens were euthanized at 10 dpi to collect spleen tissues. Then spleen samples were pooled for ChIP-Seq analysis. All procedures were performed following USDA, ARS, ADOL's Guidelines for Animal Care and Use (revised April, 2005) and the Guide for the Care and Use of Laboratory Animals published by Institute for Laboratory Animal Research (ILAR Guide) in 1996 (http://www.nap.edu/openbook.php?record_id=5140).

### ChIP and Library Construction for Solexa Sequencing Analysis

ChIP was performed using spleen samples from MDV-infected and non-infected controls birds as previously described [36]. Briefly, spleen samples were cut to small pieces (1 mm^3^) and then digested with MNase to obtain mononucleosomes for histone modification mapping. Chromatin from 30 mg spleen tissues was used for each ChIP experiment. ChIP DNA ends pulled down by H3K4me3 or H3K27me3 antibodies were repaired using End repair module (NEB, Ipswich, MA, USA) and a 3′ A was added by Taq polymerase for adaptor ligation (NEB, Ipswich, MA, USA). A pair of Solexa adaptors (Illumina, San Diego, CA, USA) was ligated to the repaired ends. PCR was performed for 17 cycles on ChIP DNA using the adaptor primers, and fragments with a length of approximately 220 bp (mononucleosome+adaptors) were isolated from the agarose gel. Cluster generation and sequencing analysis using the purified DNA was performed on the Solexa 1G Genome Analyzer (Illumina, San Diego, CA, USA) following the manufacturer's protocols. The antibodies used and the number of total tags obtained for each sample is listed in Table S6 in the supplemental material. Before further analysis of the ChIP-Seq data, the quality of the antibodies, ChIP and predicted H3K4me3 and H3K27me islands were confirmed (See Figure S8, S9, S10 in the supplemental material).

### Read mapping and summary counts

Sequence reads obtained from the Solexa Genome Analyzer were aligned to the May 2006 version of the chicken genome (galGal3) using Maq version 0.7.1 [82]. We used the default alignment policies of Maq: a maximum of two mismatches were allowed in a valid alignment and if a read aligned equally well to multiple locations in the genome, one location was chosen at random. We also removed redundant reads before further analysis to prevent amplification bias. Read counts were calculated using non-overlapping windows of 200 bp for visualization on the UCSC genome browser. For the purpose of comparisons, summary counts in each sample were normalized to per million mapped reads in the corresponding sample.

### Peak identification with SICER

Summarized read counts were subjected to peak calling with SICER [83]. The source code was modified to include support for the chicken genome. Fragment length was specified to be about 190 bp as estimated from our ChIP-Seq experiments. A window size of 200 (default) and gap size of 400 bp was used for the analysis. The E-value for estimating significant islands was set to 100.

### Merged islands

For the purposes of comparing across samples, significant islands found in similar regions of different samples were merged to obtain a consolidated list of enriched regions. The merging algorithm was as follows: significant islands in one sample we used to initialize the list. For each region *M* in the list, we searched for significant islands in another sample. In the case of an overlap between *M* and a significant island, *I*, the merged region was updated to a union of the two regions, 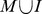. This procedure was repeated over all samples to obtain a consolidated list of merged islands.

### Island gene overlaps and genome-wide distribution

RefSeq and Ensembl gene annotations were downloaded from UCSC genome browser. Because there were only 4306 RefSeq genes in the database, we included Ensembl genes in our analysis. There are 17,858 annotated genes in the Ensembl database which include validated and predicted genes. To maximize coverage of annotated genes we combined the two databases as follows: if a genomic region was annotated with both RefSeq and Ensembl genes, we use the RefSeq gene to annotate the region. As a result of this step, we obtained a non-redundant list of 18198 genes consisting of 4306 RefSeq genes and 13892 Ensembl genes.

We searched for overlaps between merged islands and the non-redundant list of annotated genes. For H3K4me3, a significant island was annotated for a gene if it overlapped the TSS region of the gene. In the case of H3K27me3, a valid overlap constitutes a significant island overlapping the gene body. To calculate the genomic distribution of significant islands we counted all islands that overlapped with one of the following regions: promoter (TSS 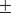 500 bp), exons, introns, 5′ UTR and 3′ UTR.

### Histone modification profiles and correlation with transcript levels

Genes were divided into ten categories based on their absolute expression. Histone modification profiles were calculated in 1 kb windows 5 kb upstream to 5 kb downstream of the gene. For reads falling within the gene body, read counts were obtained in bins of 5% of the gene length. We also obtained read count profiles in 5 bp windows for 2 kb on either side of the TSS of the genes. The read counts were finally normalized to the total number of genes in the categories and the total number of reads in the sample.

### miRNA target prediction and gene functional study

The target genes for miRNAs were predicted by miRDB (http://mirdb.org/miRDB/links.html). Gene functional study was performed by gene ontology (GO term) analysis (http://www.geneontology.org/) and PANTHER classification system (http://www.pantherdb.org/).

### Validation of ChIP, ChIP-Seq, genes and miRNAs expression results by Q-PCR

The quality of the ChIP, unique peaks found in ChIP-Seq, genes and miRNAs expression were tested by real-time RT-PCR using an iCycler iQ PCR system (Bio-Rad, Hercules, CA, USA). The real-time RT-PCR reactions were performed in a final volume of 20 µl with a QuantiTect SYBR Green PCR Kit (Qiagen, Valencia, CA, USA) according to the manufacturer's instructions. The primers for all of the genes analyzed are designed in an online primer system (http://frodo.wi.mit.edu/primer3/) and primer sequences were shown in Table S7 in the supplemental material. The expression of the following miRNAs was detected: gga-let-7i, gga-mir-130a, gga-mir-130b, gga-mir-199-2, gga-mir-1639, gga-mir-1a-2, gga-mir-124a, gga-mir-219, and gga-mir-310. The primers for the miRNAs were designed and synthesized in QIAGEN company (Qiagen, Valencia, CA, USA).

## Supporting Information

Figure S1 **Work flow of the experiments and visualization of the result on IGV (Integrative Genomics Viewer).** A. Work flow of the experiments. Infection experiments were carried out in chickens both resistant and susceptible to MD. After 10 days of infection, spleen samples were collected from infected and non-infected age-matched control group. ChIP-Seq experiments were used to generate the genome-wide H3K4me3 and H3K27me3 map. Gene expression profiles were obtained by microarray. B. Visualization of histone methylation enrichment on IGV (Integrative Genomics Viewer) after peak calling. A representative region from Chromosome 1 spanning about 489 kb was shown. The number of reads mapped to the genome in a 200 bp window was shown on the left of each track. The gene names were shown below.
(TIF)

Figure S2 **H3K4me3 enrichment in TSS and gene body regions and its relationship with gene expression.** A. H3K4me3 enrichment and its relationship with gene expression along the gene, including promoter, TSS, gene body, TTS and intergenic region in L6_3_ non-infected group. Genes are categorized into 10 groups. The expression level increased from cat1 genes to cat10 genes. To avoid the confusion caused by different length of the gene body of each gene, a relative position of each part of the gene body was shown on X-axis from 0 to 1. B. H3K4me3 enrichment and its relationship with gene expression in L6_3_ infected group. C. H3K4me3 enrichment and its relationship with gene expression in L7_2_ non-infected group. D. H3K4me3 enrichment and its relationship with gene expression in L7_2_ infected group.
(TIF)

Figure S3 **H3K27me3 enrichment in TSS and gene body regions and its relationship with gene expression.** A. H3K27me3 enrichment and its relationship with gene expression along the gene, including promoter, TSS, gene body, TTS and intergenic region in L6_3_ non-infected group. Genes are categorized into 10 groups. The expression level increased from cat1 genes to cat10 genes. To avoid the confusion caused by different length of the gene body of each gene, a relative position of each part of the gene body was shown on X-axis from 0 to 1. B. H3K27me3 enrichment and its relationship with gene expression in L6_3_ infected group. C. H3K27me3 enrichment and its relationship with gene expression in L7_2_ non-infected group. D. H3K27me3 enrichment and its relationship with gene expression in L7_2_ infected group.
(TIF)

Figure S4 **Correlation of histone modification enrichment and gene expression in TSS and gene body (GB) region.**
(TIF)

Figure S5 **H3K4me3 and H3K27me3 profile at candidate genes for MD-resistance and –susceptibility.** H3K4me3 and H3K27me3 enrichment and the expression of *CD8α* (A), *CTLA4* (B), and *IL8* (C) gene in four groups. The histone modification profile was shown in custom track in IGV. The position of the gene was indicated on the bottom of the panel. The arrow means the transcriptional direction of the gene. The gene expression analysis were done by Q-PCR. N = 4 for each group. *P<0.05, **P<0.01. Error bar = SEM.
(TIF)

Figure S6 **Correlation of miRNA expression with H3K4me3 (A) and H3K27me3 (B) enrichment.**
(TIF)

Figure S7 **Pathways analysis of the targets genes of miRNA with unique H3K27me3 enrichment in other** G **protein coupled receptors signaling pathway.** The miRNA targets were classified in PANTHER (http://www.pantherdb.org/). Dashed line: threshold line corresponds to P value of 0.05.
(TIF)

Figure S8 **Test of antibody specificity by western blot.** A western blot was performed using the human source H3K4me3 and H3K27me3 antibody in spleen samples from 2 individuals. K4: H3K4me3; K27: H3K27me3.
(TIF)

Figure S9 **Validation of ChIP quality by Q-PCR.** The quality of the ChIP was validated by quantitative PCR using primers located on the promoter region of *GAPDH* and *MyoD* respectively. The ChIP quality of H3K4me3 (A) and H3K27me3 (B) was detected by Q-PCR.
(TIF)

Figure S10 **Validation of the H3K4me3 and H3K27me3 peaks by Q-PCR.** Primers were designed based on the predicted H3K4me3 and H3K27me3 islands. The upper panel is the visulized histone methylation islands in IGV. The lower panel is the Q-PCR result showing the relative histone methylation enrichment normalized with with the region with no histone methylation islands. A. H3K4me3 island that are different between L6_3_ and L7_2_. B and C. H3K4me3 island that was identified in all samples. D. H3K27me3 island that was identified in all samples.
(TIF)

Table S1 **Genes with unique histone modification mark (H3K4me3 or H3K27me3) in different groups.**
(XLSX)

Table S2 **Gene ontology analysis of the genes with unique histone modification mark.**
(XLSX)

Table S3 **miRNAs targeted by H3K4me3.**
(XLSX)

Table S4 **miRNAs targeted by H3K27me3.**
(XLSX)

Table S5 **Significant pathways of the target genes of unique miRNAs marked by H3K27me3.**
(XLSX)

Table S6 **Tags and mapped tags after sequencing for four groups.**
(DOCX)

Table S7 **Primers used in this study.**
(DOCX)
