# Supplementary figures and images for: Histone Methylation Analysis and Pathway Predictions in Chickens after MDV Infection
Source: PLoS One. 2012 Jul 26;7(7):e41849. doi: 10.1371/journal.pone.0041849 (PMC3406056; doi:10.1371/journal.pone.0041849)

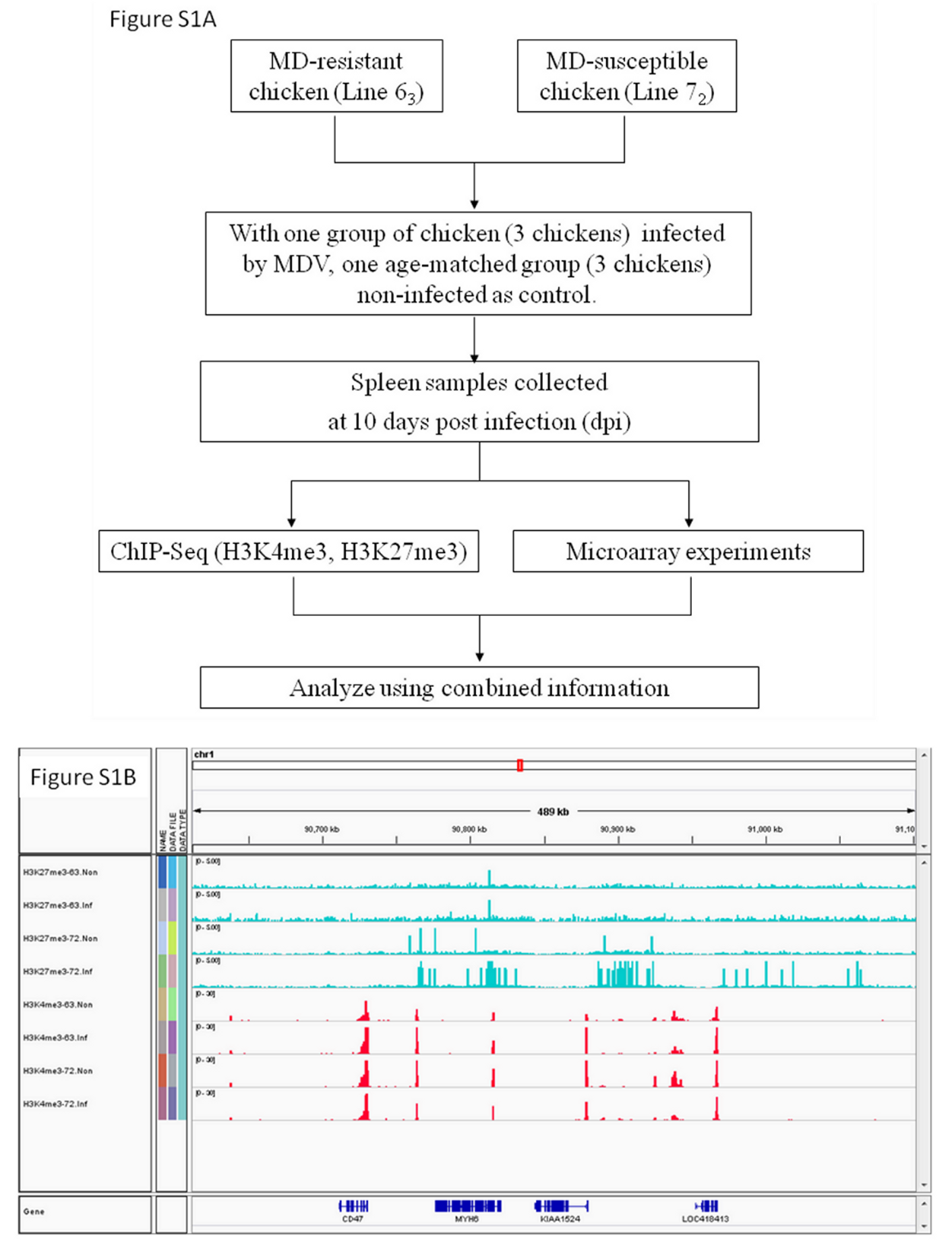

Supplement: Figure S1 — Work flow of the experiments and visualization of the result on IGV (Integrative Genomics Viewer). A. Work flow of the experiments. Infection experiments were carried out in chickens both resistant and susceptible to MD. After 10 days of infection, spleen samples were collected from infected and non-infected age-matched control group. ChIP-Seq experiments were used to generate the genome-wide H3K4me3 and H3K27me3 map. Gene expression profiles were obtained by microarray. B. Visualization of histone methylation enrichment on IGV (Integrative Genomics Viewer) after peak calling. A representative region from Chromosome 1 spanning about 489 kb was shown. The number of reads mapped to the genome in a 200 bp window was shown on the left of each track. The gene names were shown below. (TIF) [file pone.0041849.s001.tif]

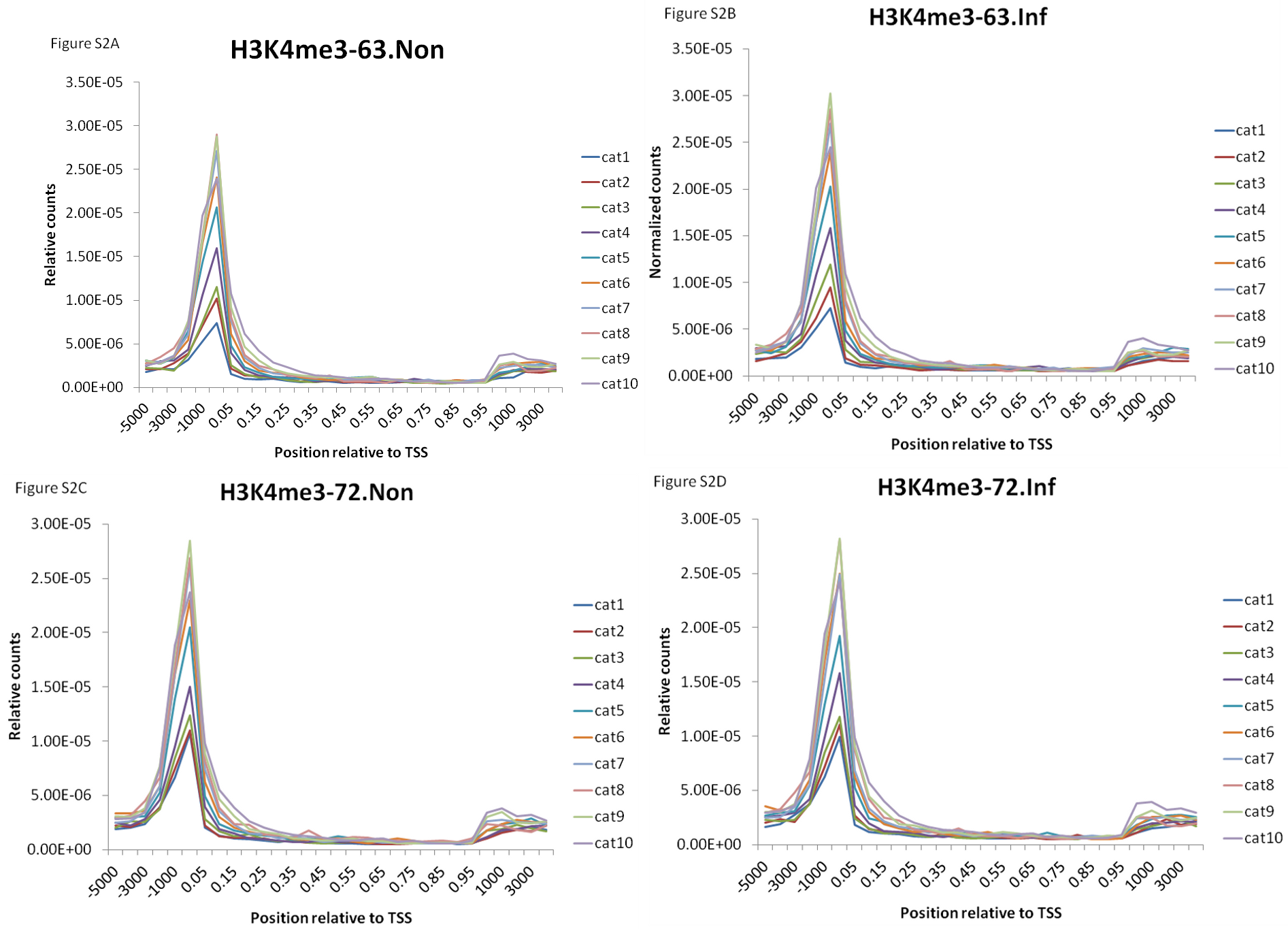

Supplement: Figure S2 — H3K4me3 enrichment in TSS and gene body regions and its relationship with gene expression. A. H3K4me3 enrichment and its relationship with gene expression along the gene, including promoter, TSS, gene body, TTS and intergenic region in L63 non-infected group. Genes are categorized into 10 groups. The expression level increased from cat1 genes to cat10 genes. To avoid the confusion caused by different length of the gene body of each gene, a relative position of each part of the gene body was shown on X-axis from 0 to 1. B. H3K4me3 enrichment and its relationship with gene expression in L63 infected group. C. H3K4me3 enrichment and its relationship with gene expression in L72 non-infected group. D. H3K4me3 enrichment and its relationship with gene expression in L72 infected group. (TIF) [file pone.0041849.s002.tif]

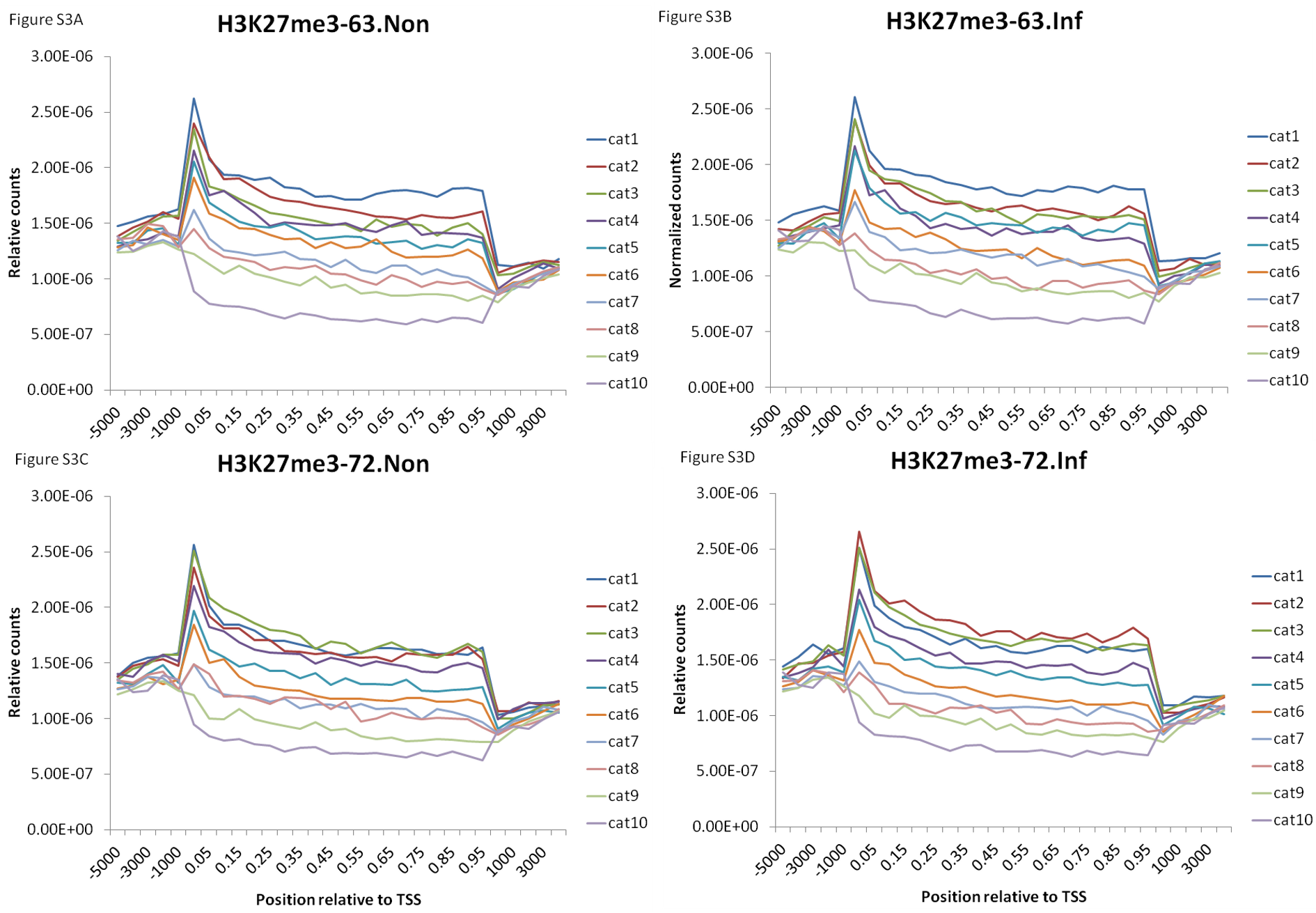

Supplement: Figure S3 — H3K27me3 enrichment in TSS and gene body regions and its relationship with gene expression. A. H3K27me3 enrichment and its relationship with gene expression along the gene, including promoter, TSS, gene body, TTS and intergenic region in L63 non-infected group. Genes are categorized into 10 groups. The expression level increased from cat1 genes to cat10 genes. To avoid the confusion caused by different length of the gene body of each gene, a relative position of each part of the gene body was shown on X-axis from 0 to 1. B. H3K27me3 enrichment and its relationship with gene expression in L63 infected group. C. H3K27me3 enrichment and its relationship with gene expression in L72 non-infected group. D. H3K27me3 enrichment and its relationship with gene expression in L72 infected group. (TIF) [file pone.0041849.s003.tif]

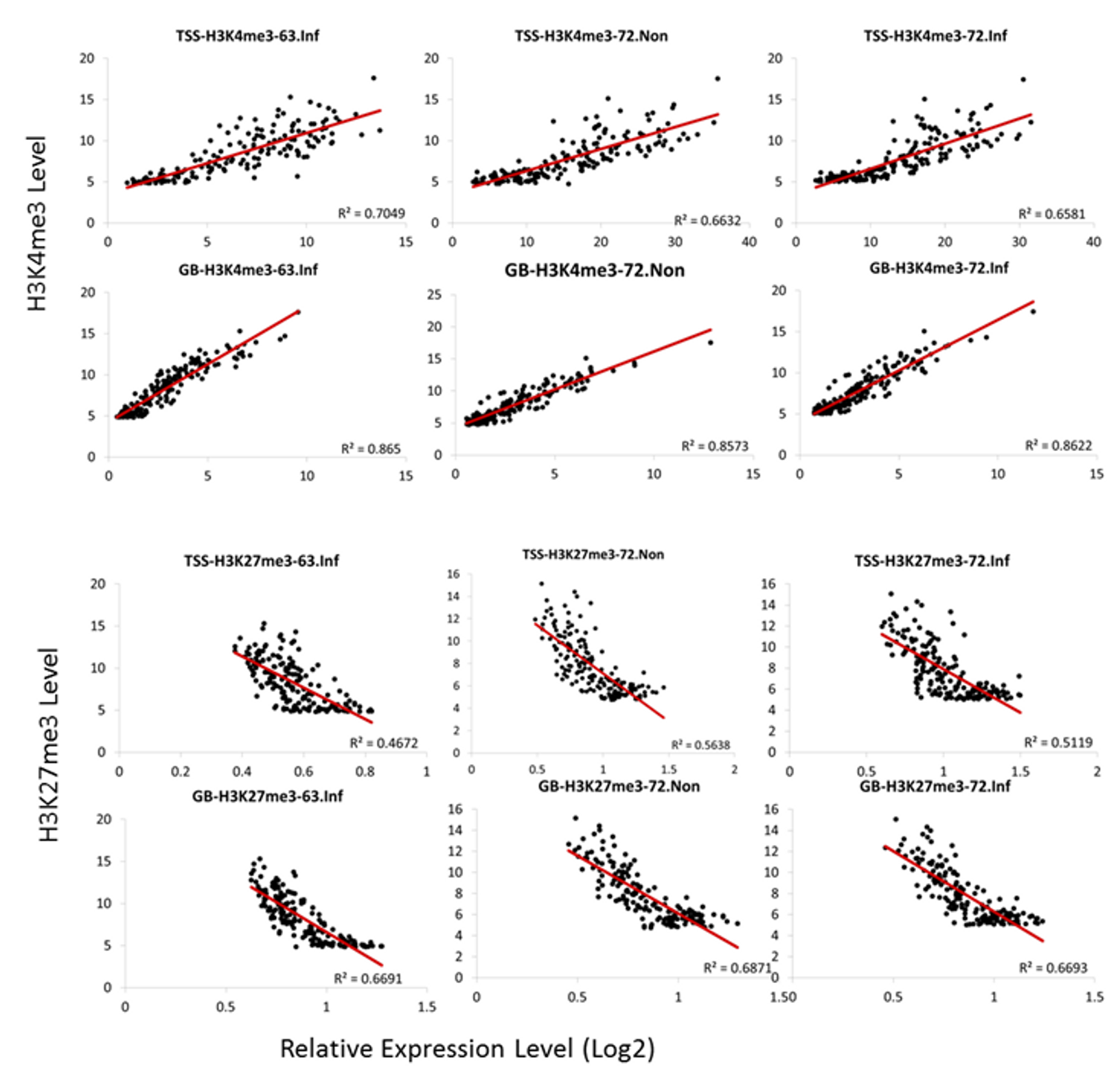

Supplement: Figure S4 — Correlation of histone modification enrichment and gene expression in TSS and gene body (GB) region. (TIF) [file pone.0041849.s004.tif]

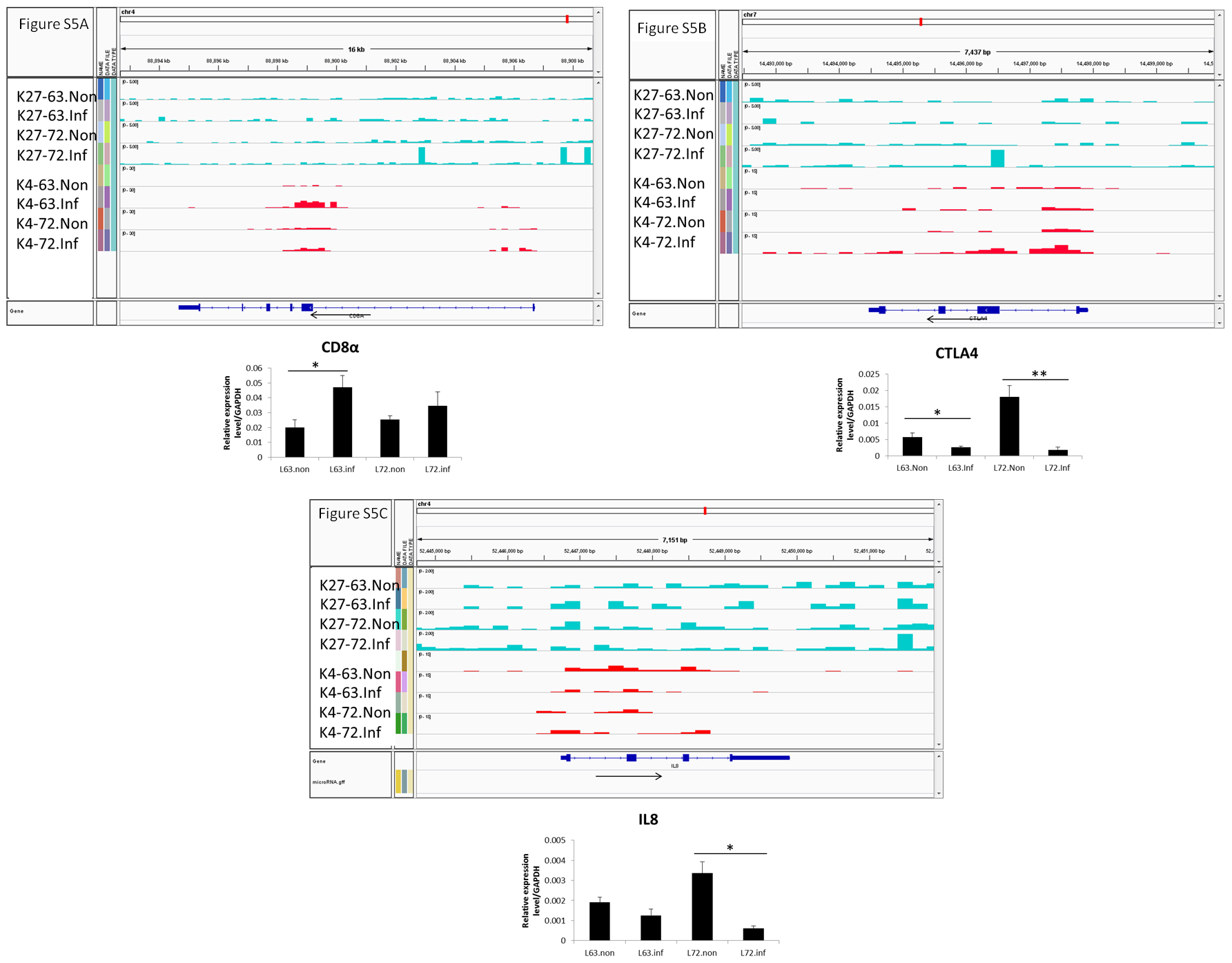

Supplement: Figure S5 — H3K4me3 and H3K27me3 profile at candidate genes for MD-resistance and –susceptibility. H3K4me3 and H3K27me3 enrichment and the expression of CD8α (A), CTLA4 (B), and IL8 (C) gene in four groups. The histone modification profile was shown in custom track in IGV. The position of the gene was indicated on the bottom of the panel. The arrow means the transcriptional direction of the gene. The gene expression analysis were done by Q-PCR. N = 4 for each group. *P<0.05, **P<0.01. Error bar = SEM. (TIF) [file pone.0041849.s005.tif]

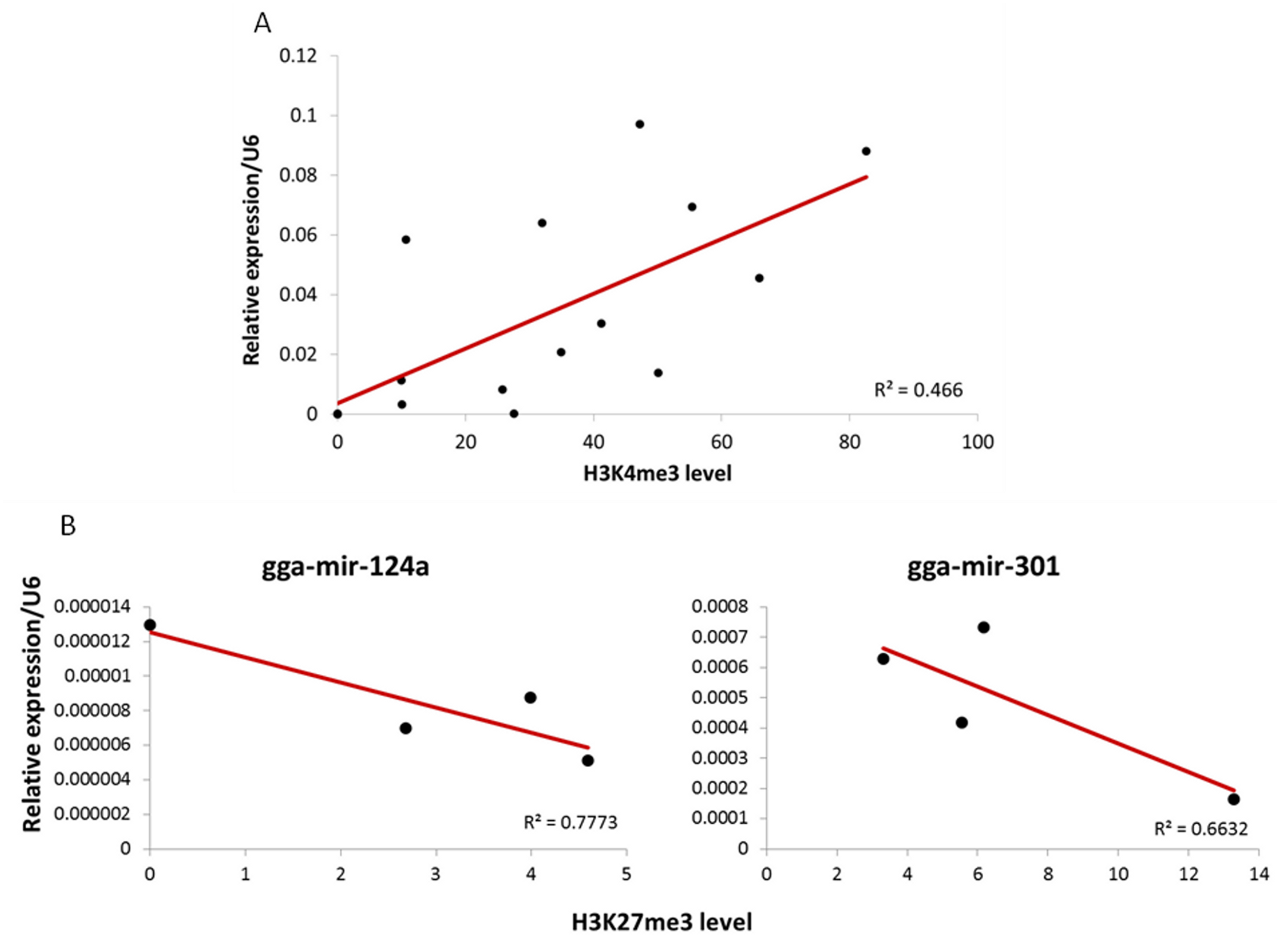

Supplement: Figure S6 — Correlation of miRNA expression with H3K4me3 (A) and H3K27me3 (B) enrichment. (TIF) [file pone.0041849.s006.tif]

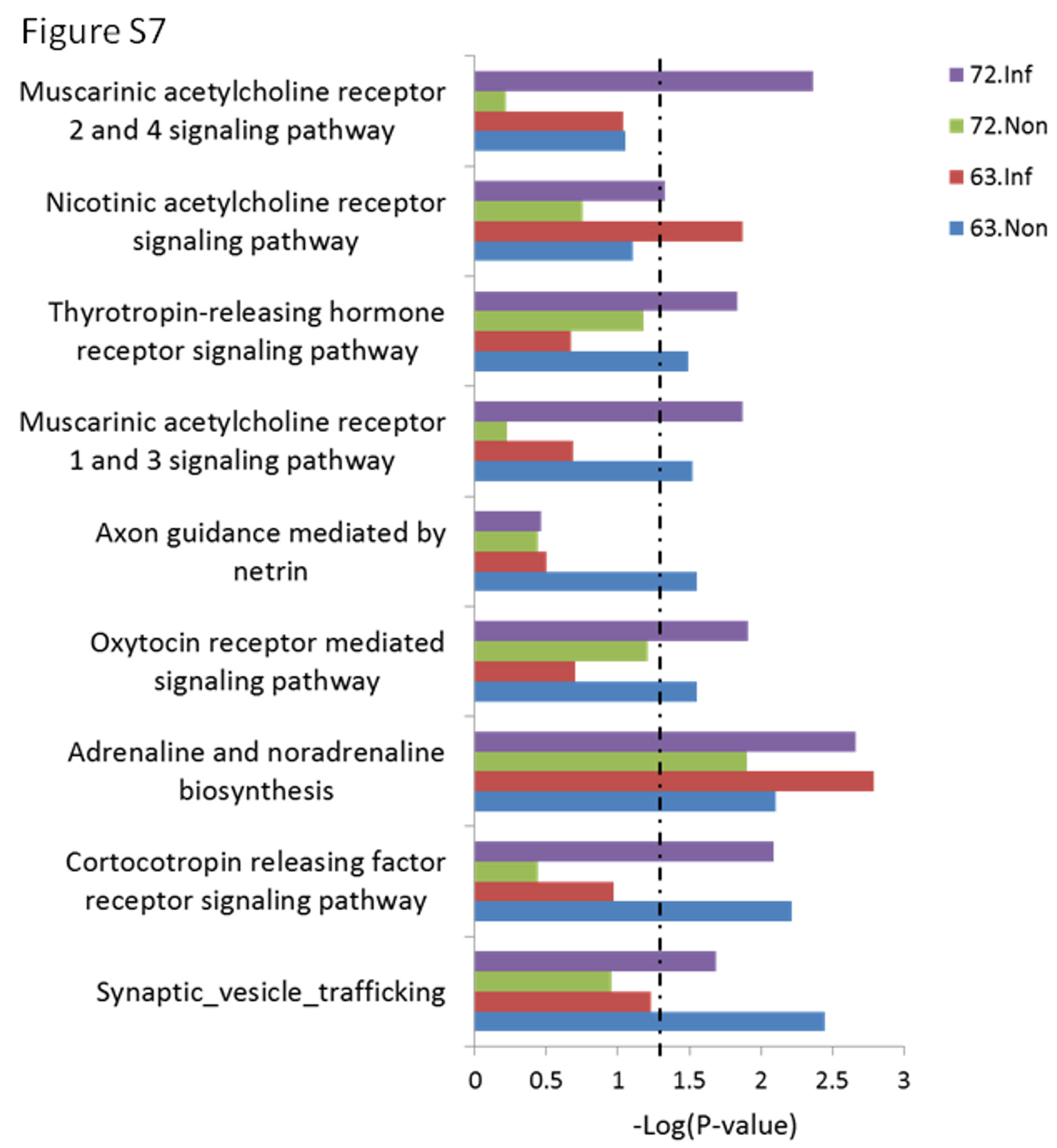

Supplement: Figure S7 — Pathways analysis of the targets genes of miRNA with unique H3K27me3 enrichment in other G protein coupled receptors signaling pathway. The miRNA targets were classified in PANTHER (http://www.pantherdb.org/). Dashed line: threshold line corresponds to P value of 0.05. (TIF) [file pone.0041849.s007.tif]

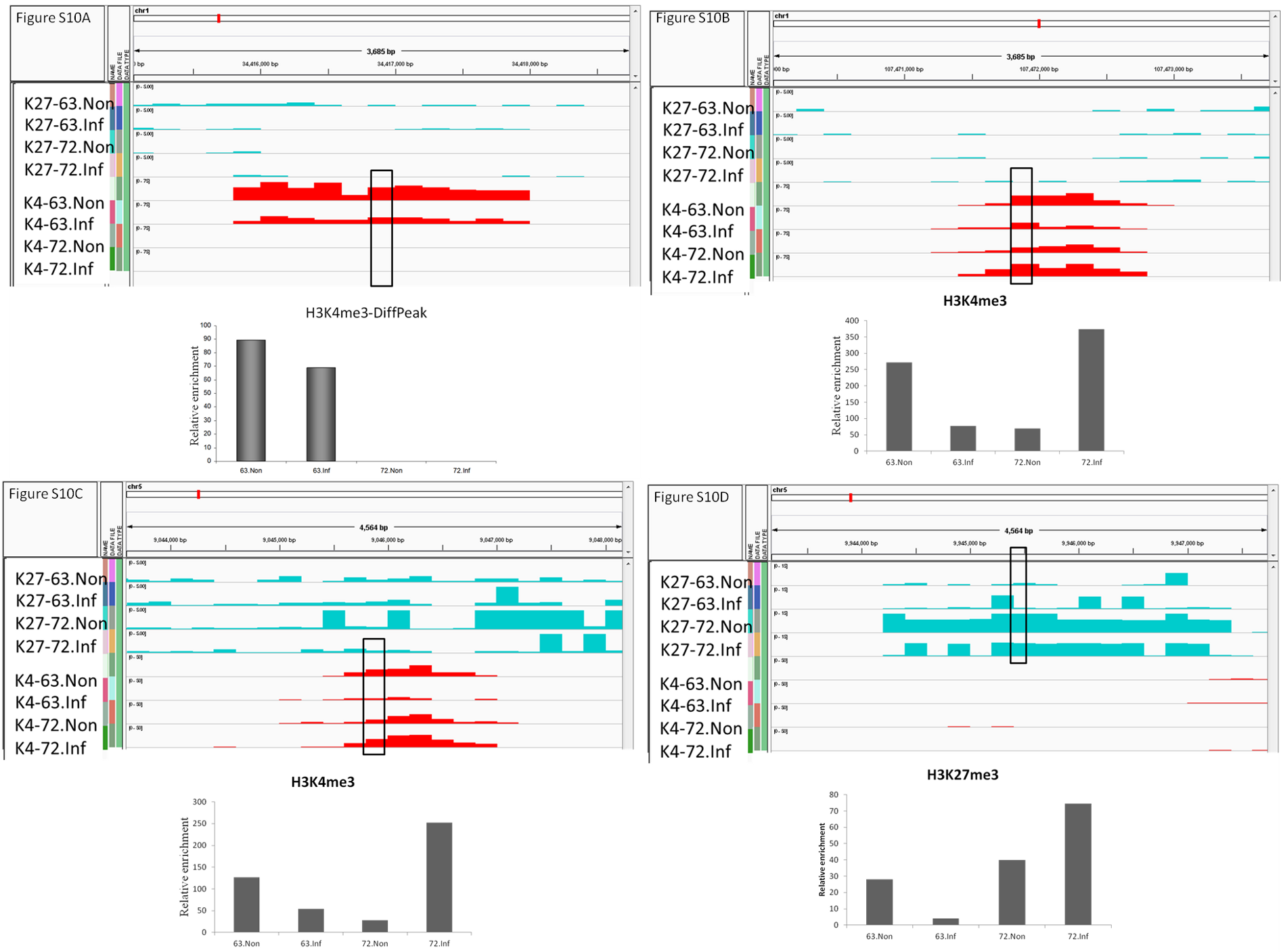

Supplement: Figure S10 — Validation of the H3K4me3 and H3K27me3 peaks by Q-PCR. Primers were designed based on the predicted H3K4me3 and H3K27me3 islands. The upper panel is the visulized histone methylation islands in IGV. The lower panel is the Q-PCR result showing the relative histone methylation enrichment normalized with with the region with no histone methylation islands. A. H3K4me3 island that are different between L63 and L72. B and C. H3K4me3 island that was identified in all samples. D. H3K27me3 island that was identified in all samples. (TIF) [file pone.0041849.s010.tif]
