# Supplementary material for: Histone Methylation Analysis and Pathway Predictions in Chickens after MDV Infection
Source: PLoS One. 2012 Jul 26;7(7):e41849. doi: 10.1371/journal.pone.0041849 (PMC3406056; doi:10.1371/journal.pone.0041849)
Supplement: Table S6 — Tags and mapped tags after sequencing for four groups. (DOCX) [file pone.0041849.s016.docx]

**Table S6. Tags and mapped tags after sequencing for four groups**

| Tissue | Antibodies | Chicken Lines | Treatments | Raw Reads | Mapped Reads | Mapped % |
| --- | --- | --- | --- | --- | --- | --- |
| Spleen | H3K4me3  (Millipore, Cat. #17-614) | 6_3_ | Inf. | 8856545 | 7663430 | 86.5 |
|  |  |  | Non. | 9691450 | 5271820 | 54.4 |
|  |  | 7_2_ | Inf. | 13205325 | 10686290 | 80.9 |
|  |  |  | Non. | 12327437 | 10717375 | 86.9 |
|  | H3K27me3  (Abcam, Cat. # ab6002) | 6_3_ | Inf. | 10116802 | 5751961 | 56.9 |
|  |  |  | Non. | 11963585 | 10646707 | 89.0 |
|  |  | 7_2_ | Inf. | 14245932 | 13499163 | 94.8 |
|  |  |  | Non. | 11592319 | 9925303 | 85.6 |
