# Supplementary material for: Histone Methylation Analysis and Pathway Predictions in Chickens after MDV Infection
Source: PLoS One. 2012 Jul 26;7(7):e41849. doi: 10.1371/journal.pone.0041849 (PMC3406056; doi:10.1371/journal.pone.0041849)
Supplement: Table S7 — Primers used in this study. (DOCX) [file pone.0041849.s017.docx]

**Table S7. Primers used in this study**

| **Genes** | **Purpose** | **GeneBank Accession Number** | **Primers** | ***Sequence*** |
| --- | --- | --- | --- | --- |
| *CTLA-4* | Gene expression | NM_001040091 | F  R | *5’- TCAAACAGACAGGCGACAAG-3’*  *5’- GGGCTAACATGGCACTGAAT-3’* |
| *CD8α* | Gene expression | NM_205235 | F  R | *5’-TTGGACGGGACCTTACAGAC-3’*  *5’-TCAGCCTGCAGGAGTGTAAA-3’* |
| *IL8* | Gene expression | NM_205498 | F  R | *5’-TGGAGCTGTCCTGGCCCTCC-3’*  *5’ GCCGCTTGGCGTCAGCTTC -3’* |
| *GAPDH* | Gene expression | NM_204305 | F  R | *5’-GAGGGTAGTGAAGGCTGCTG-3’*  *5’-ACCAGGAAACAAGCTTGACG-3’* |
| *GAPDH-ChIP* | ChIP validation |  | F  R | *5’-GTCACGTCCCAGGAGCAG-3’*  *5’-AGGACCGTGCTAATGAGGAA-3’* |
| *MyoD-ChIP* | ChIP validation |  | F  R | *5’-TTGGTGGAGATCATGCCATA-3’*  *5’-GTTGTGGGCCAGAAACAAGT-3’* |
| *K4-DiffPeak* | ChIP island validation |  | F  R | *5’-TCCTCCTTATGTGGGGAGTG-3’*  *5’-GGACCTGTACTCGCAAGCTC-3’* |
| *K4-Peak-1* | ChIP island validation |  | *F*  *R* | *5’-AGCATGCAGCTCCCTAAAAA-3’*  *5’-CCTTGGGGATAAGGAATGGT-3’* |
| *K4-Peak-2* | ChIP island validation |  | *F*  *R* | *5’-ATTTGGCTGAAAGCACCAAC-3’*  *5’-GCTTGGAGACTCCTGATTGC-3’* |
| *K4-Neg-1* | ChIP island validation  normalization |  | *F*  *R* | *5’-ATTGTGATGCCCCAAACACT-3’*  *5’-CACCATTGCATTGTCAAGGA-3’* |
| *K27-Peak-1* | ChIP island validation |  | *F*  *R* | *5’-TCAGACTGGAGACGTGATGC-3’*  *5’-GCAGCTTTCCCCTGTCAATA-3’* |
| *K27-Neg-1* | ChIP island validation  normalization |  | *F*  *R* | *5’-CCAAAGAGGCAAAACCAAAA-3’*  *5’-TCTGTCCCGTGTGTGTGATT-3’* |
